# The Key Job Demands and Resources of Nursing Staff: An Integrative Review of Reviews

**DOI:** 10.3389/fpsyg.2020.00084

**Published:** 2020-01-31

**Authors:** Sylvia Broetje, Gregor J. Jenny, Georg F. Bauer

**Affiliations:** Public & Organizational Health, Center of Salutogenesis, Epidemiology, Biostatistics and Prevention Institute, University of Zurich, Zurich, Switzerland

**Keywords:** job demands-resources model, JD-R model, nurses, integrative review, healthcare, occupational health

## Abstract

The aim of our review is to identify the key job resources and demands of nursing staff by integrating findings from previously published reviews along the lines of the JD-R model. Understanding these is highly relevant given the ever-increasing pressure in nursing work and the challenges of healthcare organizations in recruiting qualified staff. It is also an important step toward developing targeted workplace interventions. A comprehensive search of the literature identified 14 quantitative and qualitative reviews that were included in our integrative review of reviews. Thematic analysis identified three key job demands and six key job resources of nursing staff, namely work overload, lack of formal rewards, work-life interference, supervisor support, fair and authentic management, transformational leadership, interpersonal relations, autonomy and professional resources. Our results corroborate findings from previous reviews, expand the relevance and generalizability by considering a broader range of relevant health-related and motivational outcomes, and highlight the importance of leadership practices in nursing.

## Introduction

Nurses and their employers are faced with substantial challenges. Nurses have been found to experience considerable strain at work that is related to high workloads, emotional demands, shift work, or understaffing, while healthcare organizations are struggling to attract and retain qualified staff. Studies show that approximately one third of nurses in Europe and the United States feel burnt out (Aiken et al., 2012). Thirty three percent of nurses want to leave their current employer within the next year due to job dissatisfaction and 9% intend to leave the profession altogether (Heinen et al., 2013).

Across the OECD, the health and social work sector constitutes ~10% of employment and is steadily growing. Between 2000 and 2015, employment grew in this sector by a mean of 42%, surpassing that of the services sector. While recent prognoses predict a less severe nurses shortage than originally anticipated, increasing the retention rates of nurses remains an ongoing challenge (OECD, 2017).

A group that is characterized by particularly high strain are nursing staff working in long-term care, such as nursing homes. German statistics show that the number of sick days of nursing home nurses (24.1 days) substantially surpasses that of acute care nurses (19.3 days), which again surpasses that of the general working population (16.1 days). At the same time, the availability of occupational health programs is much lower for this group (Kliner et al., 2017). Nursing work is not only characterized by high demands, however, it offers unique rewards as well (Sinclair et al., 2015). With this integrative review of reviews, we intend to contribute to the understanding of the most important workplace antecedents that affect health-related and motivational outcomes, such as exhaustion, job retention or job satisfaction, in nurses.

**Figure 1:**
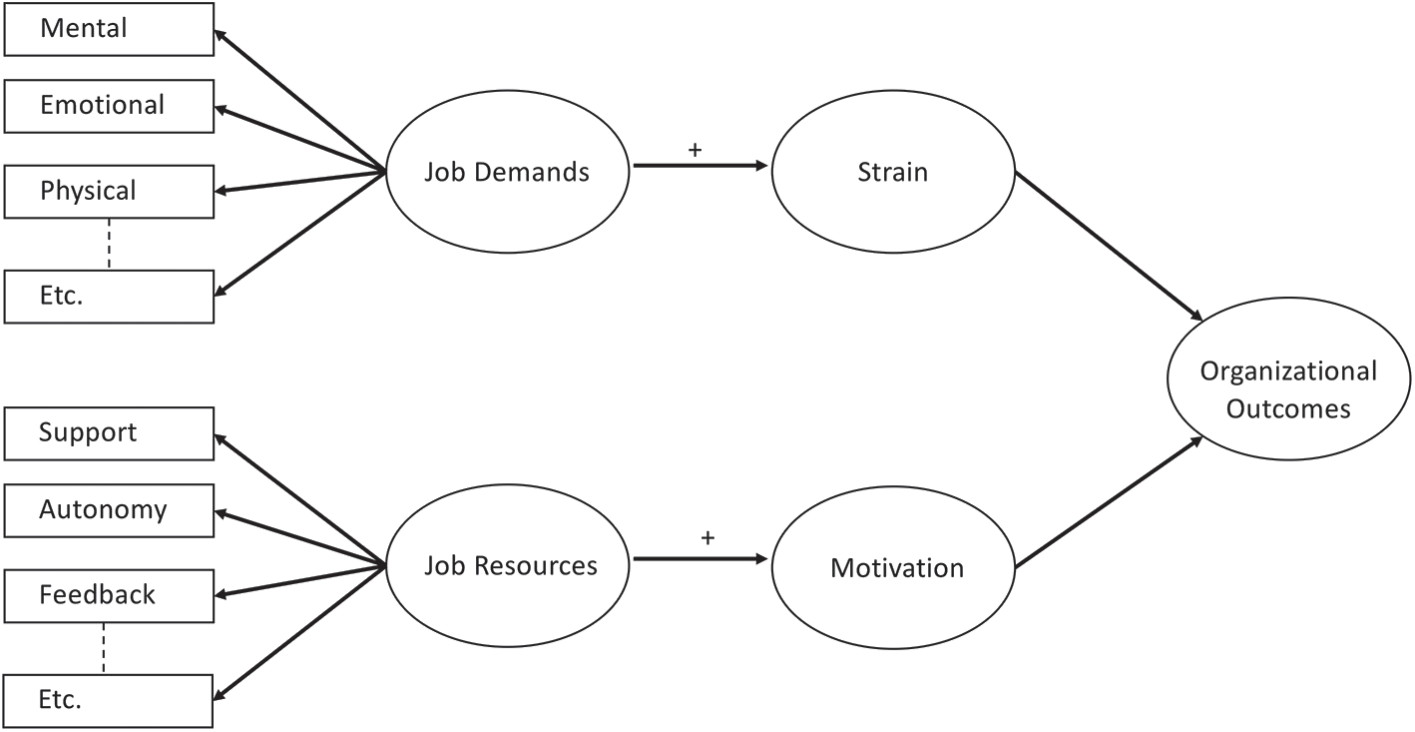
Simplified Job Demands-Resources Model, adapted from Bakker and Demerouti (2007).

The Job Demands-Resources (JD-R) model by Demerouti et al. (2001) has been established as a framework to study and address workplace characteristics. The JD-R model proposes two main pathways, linking job resources to motivational outcomes such as work engagement, and linking high levels of job demands to strain (see Figure 1, adapted from Bakker and Demerouti, 2007).

Job demands are defined as “those physical, social, or organizational aspects of the job that require sustained physical or mental effort and are therefore associated with certain physiological and psychological costs” (Demerouti et al., 2001, p. 501). Job resources, on the other hand, refer to “those physical, psychological, social, or organizational aspects of the job that may do any of the following: (a) be functional in achieving work goals; (b) reduce job demands and the associated physiological and psychological costs; (c) stimulate personal growth and development” (Demerouti et al., 2001, p. 501). These relationships proposed by the model have found substantial support in empirical studies (see Bakker and Demerouti, 2007 for an overview). However, Bakker and Demerouti (2017) also point to several issues that warrant further examination. These include possible direct links between job resources and demands as well as the importance of assessing demands and resources not only on the level of the individual, but also on the team and organizational level. Bakker and Demerouti also propose that job demands may need to be viewed separately as hindrance demands and challenge demands (LePine et al., 2005). While both require the exertion of effort, the latter bear the reward of personal growth and achievement. Their appraisal as one or the other may be dependent on the context. The authors also call for further examination of the underlying psychological or physiological mechanisms involved in the health impairment and motivational process, where the JD-R model currently relies on external theories.

A number of job resources and demands have been established for the general working population. Bakker and Demerouti (2007) name mental, emotional and physical demands as the main job demands and a study by Bakker et al. (2005) includes also work overload and work-home interference as demands relevant for most people's work environment. Brauchli et al. (2015), in a study with more than 2,000 employees in different industries, including hospitals, identified the following generalizable job demands: quantitative demands (work interruption and time pressure), qualitative overload and uncertainty at work. With regards to job resources, Bakker and Demerouti (2007) emphasize support, autonomy, and feedback, to which Bakker et al. (2005) add quality of the relationship with the supervisor. Brauchli et al. (2015) identified manager behavior (supportive leadership, interpersonal justice, manager support, manager appreciation), peer behavior (peer support, peer appreciation) and task-related resources (task identity, job control) as generalizable job resources.

The JD-R model has also been applied in the nursing context, most notably by McVicar (2016) and by Keyko et al. (2016). McVicar (2016), based on the finding that job stress and job satisfaction are two highly correlated and inversely related constructs, conducted a scoping review that compared the antecedents of job stress and job satisfaction in nurses. His review included 24 quantitative, three qualitative and one mixed-methods study published between 2000 and 2013, and relevant workplace antecedents of job stress and job satisfaction were categorized into job demands and resources by qualitative analysis. The job demands most consistently related to both job stress and job satisfaction across the review period were work pressure and emotional demands. Work pressure included aspects like workload, staffing and physical resources, while emotional demands included aspects such as dealing with death and dying, interactions with patients and relatives and responsibility associated with care. Another, though less relevant, demand was work-life interference, especially with regards to shift work. The job resources most commonly related to both job stress and job satisfaction were interpersonal and social relations, management and supervision, decision latitude and task significance. Management and supervision comprised the aspects of support and leadership style. The resources of effort-reward and task variety were found to be only associated with job satisfaction but not with job stress. Effort reward included aspects like pay, reward, and job security. In addition to identifying the job demands and resources, McVicar also noted chronological trends, pointing out that the emotional demands of dealing with patients and relatives and work-life inference through shift work were only observed in the second half of the time period analyzed, that is from 2007 to 2013. McVicar's review demonstrates overlap between the antecedents to the two examined outcomes job stress and job satisfaction.

The outcome of interest in Keyko et al. (2016) systematic review was work engagement of nurses. They included 15 quantitative, one qualitative and two mixed-methods studies published until 2013 in their content analysis and identified a total of 77 influencing factors which they grouped into six categories: Organizational climate (leadership, structural empowerment), job resources (interpersonal and social relations, organizational, organization of work and tasks), professional resources (professional practice environment, autonomy, role and identity, professional practice and development), personal resources (psychological, relational, skill), job demands (work pressure, physical and mental demands, emotional demands, adverse environment) and demographic factors. Based on their findings, Keyko et al. present their Nursing Job Demands-Resources model for work engagement in professional nursing practice. Of note is that they place the organizational climate outside of and prior to resources, thereby highlighting the importance of organizational aspects as a “precursor to operational resources” (Keyko et al., 2016, p. 159). Furthermore, Keyko et al., emphasize the impact of professional resources by including them as a distinct resources category in their model.

Beyond these reviews specifically applying the JD-R model, a large body of literature exists examining workplace antecedents to motivational and health-related outcomes in nurses. For example, Germain and Cummings (2010) examined factors that influence nurse performance and identified 25 factors grouped into five categories (autonomy working relationships, access to resources, individual nurse characteristics and leadership practices). Bernal et al. (2015) reviewed the effects of different psychosocial risk factors (demands-control, effort-reward and social support) on musculoskeletal disease in nurses. And Daouk-Öyry et al. (2014) examined the antecedents of turnover and absenteeism and identified 91 antecedent variables for turnover and 29 for absenteeism, grouped into five main factors (individual, interpersonal, job, organizational, and national). This glimpse at the current literature illustrates the considerable interest in understanding factors influencing nurses' work motivation and health. At the same time, the current reviews are focused on single outcomes only and organize resourceful and demanding work aspects in very different ways, making it difficult to integrate and apply the findings across reviews.

With this integrative review of reviews we want to address the following research question: What are the most important workplace antecedents of nurses that are relevant across a broad range of health-related and motivational outcomes, and how can they be categorized into demands and resources? The JD-R model is uniquely suited to integrating a wide range of findings from different studies. We want to build upon and go beyond the work of McVicar (2016) and Keyko et al. (2016) by considering a broader range of outcomes, based on the health-impairment and the motivational processes in the JD-R model as well as indicators for performance and retention of nurses commonly considered in the literature. These include, among others, motivation, exhaustion, stress, work ability, absenteeism, or turnover. The aim of our study is hence to identify the key job resources and demands of nursing staff by integrating findings from previously published review studies along the lines of the JD-R model.

## Methods

### Integrative Review and Quality Appraisal

As calls for evidence-based practice in healthcare and the combination of findings from different individual studies have increased in the late twentieth century, several kinds of review methodologies and of related quality criteria have been developed (Grant and Booth, 2009). Most review approaches allow to include either quantitative or qualitative studies exclusively and some necessitate very similar research questions and study designs. Most of the associated quality appraisal tools evaluate the quality of individual studies to be included in a systematic review or meta-analysis, but few tools exist to assess the quality of reviews themselves. One such example is the AMSTAR 2 (Shea et al., 2017) which, however, focuses on the assessment of randomized and non-randomized clinical trials.

To execute our own review, we chose to conduct an integrative review of reviews. Integrative reviews provide a greater degree of flexibility than other review approaches (Whittemore and Knafl, 2005; Souza et al., 2010) and are well-established in the field of nursing research. They can be applied to a broad range of questions and permit the simultaneous inclusion of quantitative and qualitative findings. However, this flexibility can come at the price of decreased rigor. Whittemore and Knafl (2005) have put forth recommendations aimed at ensuring a high level of quality in integrative reviews and emphasize the importance of a well-specified research question, comprehensive literature search, assessment of study quality and explicit and systematic analytic approaches. To address the quality of the included reviews, we adapted the recommendations by Whittemore and Knafl and report on the relevant aspects that could be inferred from the included publications (research question, search approach, method of quality appraisal, analysis method) below.

### Inclusion and Exclusion Criteria

We set the following inclusion and exclusion criteria before conducting our literature search. Due to the breadth of outcomes considered in our integrative review, we limited our search to review articles only, resulting in an integrative review of reviews. Both quantitative and qualitative reviews were included if they performed a synthesis of their findings, rather than being merely narrative. The reviews had to examine psychosocial workplace antecedents to one or more of the outcomes discussed above and specified in Table 1. Again, based on the breadth of the included outcomes and in order to enhance the generalizability of our findings, we included only reviews that were focused on nursing staff in general care settings, both in acute care hospitals and in nursing homes. Like McVicar (2016), we excluded reviews that were focused on highly specialized nursing settings, such as hematology or intensive care. Reviews on both fully licensed nurses as well as on nursing assistants were included, based on the same reasoning that they share most workplace characteristics. We excluded reviews that focused on recent nursing graduates, due to the well-documented crisis they often experience as they transition into their professional role (Kramer, 1974; Duchscher, 2009). Reviews focusing on nurse managers were also not considered, as their day-to-day activities involve many responsibilities involving management, finances, and human resources (Kleinman, 2003), which deviate from those of typical staff nurses. Lastly, we limited our search to reviews published from the year 2000 onward, as nurses' perceptions and sources of job stress and job satisfaction have been found to change over time (McVicar, 2016), and we can assume this to be the case for other outcomes as well. We included reviews if at least two thirds of the individual studies included therein where also from 2000 onward, to ensure the currentness of the data.

**Table 1:** Search strategy.

**Databases**	**Searched outcomes**	**Inclusion criteria**
CINAHL PsycINFO MEDLINE Cochrane Library Peer-reviewed publications January 2000 –December 2018 English or German	(Positive health) Engagement Enjoyment Job satisfaction Motivation (Negative health) Burnout Disease^1^ Disorder^1^ Exhaustion Health complaints Insomnia Pain^1^ Strain Stress (Performance) Absenteeism Workability/work ability Work performance (Retention) Attrition Commitment Intention to leave Intention to stay Retention Turnover ^1^Broad search terms were specified with the Boolean expression *AND (work OR job)*	Quantitative or qualitative reviews Examination of antecedents/ predictors/determinants/ contributing factors to the listed outcomes Samples: registered nurses, nursing assistants or nursing aides Settings: general adult nursing care in hospitals or nursing homes
		**Exclusion criteria**
		Specific nursing samples, such as nursing students or nurse managers Nursing staff working in specialty care settings, such as ICU or hematology Examination of the variable in a different context, such as intervention evaluation, effects on patient care rather than on nurses, not examining antecedents on the workplace-level Meta-reviews More than a third of the studies included in the review date from before 2000 Qualitative findings described but not synthesized Poor quality
Results database search after exclusion of duplicates		104
Results manual search		3
**Total identified studies**		**107**
Removed based on title or abstract		60
Removed after in-depth reading		33
**Final selection of included articles**		**14**

### Search Strategy

We conducted a comprehensive literature search in the databases PsycINFO, CINAHL, MEDLINE, and Cochrane Library for publications in peer-reviewed journals in English or German published between 2000 and 2018. The database search was supplemented by studies identified based on references in the retrieved literature.

After combining the results from our searches and removing duplicates, 104 publications were identified and their titles and abstracts screened by the first author. Sixty Papers were excluded at this stage based on: focusing on specific nursing samples, the most common ones being nursing students or recent graduates, nurse managers, nurse practitioners and nurses working in specialty care settings such as emergency care, ICU or hematology (40), not being reviews (6), examining the variables of interest from a different angle, for example the effects of resilience on burnout or the financial costs of nursing turnover (5), reviewing the effects of interventions (4), focusing on the effects of nursing care on patients rather than the effects of workplace attributes on nurses (3), being in a language other than English or German (1), or being a meta-review (1). Thirty three papers were read in-depth but excluded because either they contained more than one third individual studies from before 2000 (9), did not address workplace antecedents (9), performed no synthesis of their findings (8), looked at prevalence rates only (3), were not peer-reviewed publications (2), or focused on nurses working in a specialty setting (1). One review was excluded as being of poor quality based on providing no information about the analysis approach used. In addition to the remaining 11 publications, hand searching of the literature identified three additional suitable publications.

### Data Extraction and Analysis

In order to integrate the findings from the included review papers, thematic analysis using MAXQDA 2018 (VERBI Software, 2017) was performed to identify and organize the most common workplace-level antecedents that affect health-related and motivational outcomes in nurses. We selected thematic analysis as a flexible and useful research tool for “identifying, analyzing and reporting patterns (themes) within data” (Braun and Clarke, 2006, p. 79) and followed Browne and Clarke's recommended sequence of analysis steps. We began by coding the relevant antecedents from each publication. An antecedent was considered relevant if it had either significantly predicted the outcome (in a meta-analysis) or was identified as a main contributor in a qualitative analysis (which was the case in most of the included reviews). If a broader category was listed as an antecedent (for example demands or organizational aspects), then we used the detailed description provided to code the specific meanings of that category. We coded our extracted data twice. In the first round of coding we developed and refined the codes and in the second round we applied the final set of codes to all data. Codes were then grouped inductively based on similarity and grouped by themes. Those were then organized into the two categories job demands and job resources. Nine themes emerged from our analysis, three job demands and six job resources.

## Results

### Search Results

Fourteen reviews met our criteria and were included in our final analysis. Their details are displayed in Table 2. Both the health-impairment and the motivational axis of the JD-R model were represented. The outcomes most commonly studied were job satisfaction and turnover. The included 14 reviews were published between 2007 and 2018. Three of them included individual studies from before 2000. Two of the reviews were meta-analyses and one followed a quantitative approach by extracting significant findings. The remaining 11 reviews performed a qualitative analysis, most commonly thematic analysis. The authors typically combined a database search and a manual literature search. All 14 reviews were published in English. For 10 of the reviews, the literature search was conducted for English-language publications only, while four included other languages as well (Spanish, French, Persian, Chinese); however broad geographic areas were represented in the reviews. Most of the reviews addressed the quality of their included studies in a meaningful way. The vast majority focused on staff nurses working in hospitals and only two reviews specifically indicated that a subset of their included data stemmed from nurses working in nursing homes or long-term care facilities, while one specified that it included studies with nursing assistants. It should be noted, however, that the term “nurse” is likely not used equally across the broad range of geographies represented in our review. In some places it may refer to fully licensed staff only, while in others may also refer to staff with lower-level qualifications.

**Table 2:** Characteristics of the included reviews.

**Author(s), year journal**	**Aim(s)/research question(s)**	**Search and criteria** **M: Method** **Y: Years** **L: Languages** **S: Sample** **O: Other information provided**	**Studies included**	**Quality assessment of included studies**	**Geography of included studies**	**Analysis method**	**Reported antecedents**
Bernal et al. (2015), International Journal of Nursing Studies	Estimate the association between psychosocial risk factors in the workplace and musculoskeletal disorders in nurses and aides	M: Database search, 2 reviewers Y: 2001–2014 L: English, Spanish S: Hospital nurses and nursing aides O: Observational studies	17: all quantitative	Adapted version of the Standardized Quality Scale developed by van der Windt et al. (2000). Study details provided	Iran (3), China (2), Denmark (2), Greece (2), United States (2), Australia (1), Brazil (1), Germany (1), Japan (1), Netherlands (1), Mixed (1: Netherlands/Greece)	Random-effects meta-analysis and heterogeneity analysis	• High psychosocial demands–low job control • Effort-reward imbalance • Low social support
Cicolini et al. (2014), Journal of Nursing Management	Examine the relationship between nurse empowerment and job satisfaction in the nursing work environment	M: Database and website search, 2 reviewers Y: 1998–2012 L: English S: Staff nurses O: Quantitative or qualitative studies. Studies using CWEQ or CWEQ-II for measuring structural empowerment and studies using PES for measuring psychological empowerment	12: all quantitative	Quality Assessment and Validity Tool for Correlational Studies' adapted from previous systematic reviews (Cummings and Estabrooks, 2003) Study details provided	Canada (7), China (3), Italy (1), Mixed (1: England Malaysia)	Narrative synthesis	• Structural empowerment
Coomber and Barriball (2007), International Journal of Nursing Studies	Explore the impact of job satisfaction components on intent to leave and turnover in order to identify the most influential factors	M: Database search Y: 1997–2004 L: Not specified S: General adult care hospital staff nurses O: Primary or secondary research	9 studies: 7 quantitative, 1 of which was a meta-analysis, 2 mixed-methods studies	Not specified Study details provided	United States (4), Taiwan (3), Australia (1), Singapore (1)	Thematic content analysis	• Leadership • Educational attainment • Pay • Stress
Cowden et al. (2011), Journal of Nursing Management	Examine the relationship between managers' leadership practices and staff nurses' intent to stay in their current position	M: Database and manual search, 2 reviewers Y: 1985–201 L: English S: Staff nurses or staff nurses and their managers O: Quantitative and qualitative studies	23: 22 quantitative, 1 qualitative	Quantitative studies: adaptation of quality assessment tool for correlational studies by Cummings and Estabrooks (2003); qualitative study: Critical Appraisal Skills Programme by Lewin et al. (2009) Study details provided	United States (15), Canada (4), Australia (1), Germany (1), Jordan (1), Taiwan (1)	Extraction of significant findings	• Transformational leadership • Supportive work environments
Daouk-Öyry et al. (2014), International Journal of Nursing Studies	Develop an integrative multilevel framework that optimizes understanding of absenteeism and turnover among hospital nurses	M: Database search Y: 2007–2013 L: English S: Hospital nurses O: Peer-reviewed, quantitative and qualitative, three reviewers	41: 33 quantitative, 4 qualitative, 4 mixed-methods	Not specified Summary tables of select study characteristics	United States (17), Netherlands (4), Japan (3), Sweden (3), UK (2), Australia (1), Belgium (1), Brazil (1), Canada (1), Finland (1), Jordan (1), Kuwait (1), New Zealand (1), South Africa (1), Spain (1), Taiwan (2)	Content analysis Two reviewers with calculation of inter-rater agreement Table with detailed information on themes and sources	91 antecedent variables for turnover and 29 antecedent variables for absenteeism, grouped into 11 categories and 5 main factors:
							• Individual • Demographics • Personal characteristics • Job attitudes • Health and wellbeing • Interpersonal • Management style • Relationships • Job • Job demand • Job control • Organizational • HR practices • Structure • National • Labor supply • Legislation
García-Sierra et al. (2016), Journal of Nursing Management	Review empirical research about work engagement in nursing and synthesize the findings to better understand this construct	M: Database and manual search Y: 1990–2013 L: English, French or Spanish S: Staff nurses O: Empirical studies, published in a scientific journal	27: 24 quantitative, 3 qualitative	Used own criteria Study details provided	Canada (6), Australia (3), Belgium (3), United States (3), Spain (2), China (1), Ireland (1), Israel (1), Italy (1), Malaysia (1), Netherlands (1), Norway (1), Portugal (1), Uganda (1), Mixed (1: Australia United States)	Thematic analysis	• Organizational antecedents • Areas of work life • Structural empowerment • Social support • Individual antecedents • Personal traits • Professional characteristics • Family issues • Work orientation • Impact of nurse managers
Germain and Cummings (2010), Journal of Nursing Management	Explore leadership factors and behaviors that influence nurse performance and nurse performance motivation	M: Database and manual search, also searched for relevant research reports on Association websites Y: 1996–2007 L: English S: – O: Peer-reviewed, empirical quantitative or qualitative studies, 2nd reviewer assessed a subset of the results	8: all quantitative	Quality Assessment and Validation Tool for Correlational Studies (Cummings and Estabrooks, 2003) and the Effective Public Health Practice Quality Assessment Tool (McMaster University School of Nursing, 2003) Study details provided	Canada (4), US (3), Singapore (1)	Content analysis	Five categories with 25 factors: • Autonomy • Work empowerment • Structural empowerment • Empowerment • Using knowledge and skills • Autonomy • Working relationships • Communication • Informal and formal power • Clearly defined nursing roles and responsibilities • Trust and respect
							• Fair and respectful practices • Raise workload concerns • Access to resources • Managing the unit • Managing resources • Individual nurse characteristics • Ambiguity tolerance • Hardiness • Leadership practices • Enabling the heart • Modeling the way • Challenging the process • Encouraging the heart • Inspiring a shared vision • Leadership: building, coaching and mentoring
Hayes et al. (2010), Journal of Nursing Management	Explore the common factors contributing to nurse job satisfaction in the acute hospital setting	M: Database search Y: 2004–2009 L: English S: Nurses working in the acute care hospital setting	17 Studies: 12 quantitative, 2 qualitative, 1 mixed-methods, 2 development of an instrument	Not specified Study details provided	US (5), Canada (3), Australia (2), China (1), Ireland (1), Italy (1), Jordan (1), Norway (1), South Korea (1), UK (1)	Not specified Table with detailed information on clusters and sources	This review identified 44 factors in three clusters (intra-, inter- and extra- personal): • Intra-personal • Age • Education • Experience •… • Inter-personal • Autonomy • Interactions • Professional status • Relationships • Task requirements • Work life interference •… • Extra-personal • Organizational policies • Pay • Workload •…
Hayes et al. (2012), International Journal of Nursing Studies	Examine recent findings related to the issue of nursing turnover and its causes and consequences	M: Database and manual search Y: from 2006 onward L: English S: Nurses and nursing aides working in	51: all quantitative	Not specified Study details provided	Canada (7), United States (5), Australia (3), Europe (2), Taiwan (2), Belgium (1), China (1), Finland (1), Ireland (1), Japan (1), Korea (1), Macao (1),	Integrative approach	• Organizational factors • Workload, stress and burnout • Management style • Empowerment
		hospital, long-term or community care			South Africa (1), UK (1), Not specified (23)		• Role perceptions • Individual factors • Career advancement and pay benefits
Keyko et al. (2016), International Journal of Nursing Studies	Understanding influencing factors on and outcomes of work engagement in professional nursing practice	M: Database and manual search, second reviewer assessed subset of results Y: Until 2013 L: English S: Registered nurses in direct-care positions O: Peer-reviewed, quantitative and qualitative studies	18: 15 quantitative, 1 qualitative, 2 mixed-methods	Quantitative studies: adaptation of the tool by Cummings and Estabrooks (2003); qualitative study: Critical Skills Appraisal Programme Study details provided	Canada (7), USA (7), Australia (1), China (1), Iran (1), Taiwan (1)	Content analysis based on the JD-R framework	77 influencing factors grouped into six categories: • Organizational Climate • Leadership • Structural empowerment • Job Resources • Interpersonal and social relations • Organizational • Organization of work and tasks • Professional Resources • Professional practice environment • Autonomy • Role and identity • Professional practice and development • Personal Resources • Psychological • Relational • Skill • Job Demands • Work pressure • Physical and mental demands • Emotional demands • Adverse environment • Demographic factors
McVicar (2016), Journal of Nursing Management	Identify core common antecedents of job stress and job satisfaction in nurses	M: Network and database search Y: 2000–2013 L: English S: Nurses in clinical practice that were not entirely from one specialty	27 studies with 28 datasets: 24 quantitative, 3 qualitative, 1 mixed-methods	Appraisal framework by Brown et al. (2013)	United States (3), Canada (2), Iceland (2), Italy (2), Taiwan (2), Australia (1), Belgium (1), China (1), Germany (1), Iran (1), Ireland (1), Jordan (1), Netherlands (1), Norway (1), Singapore (1), South Korea (1), UK (1), Mixed (3: Japan South Korea Thailand United States, Kenya Tanzania Uganda, Sweden Norway), Not specified (1)	Quantitative studies: Significant antecedents (*p* ≤ 0.05) were included or top 5 antecedents when ranked means were reported Qualitative studies: Major themes Antecedents were clustered and fit onto the JD-R framework, which was extended where necessary.	• Interpersonal and Social Relations (Co-working, Interpersonal relationships and professional status) • Management and supervision (Support, Leadership style) • Decision Latitude • Effort-reward (Financial reward, Job professional development opportunities, Job security)
							• Task significance (Role ambiguity) • Task variety (Job content routinization) • Work Pressure (Workload time staffing, Physical resources) • Work-Life Interference (Work–family Conflict, Shift working) • Emotional Demands (Dealing with death and dying, Interaction with patients and relatives, Responsibility associated with care commitment)
Utriainen and Kyngäs (2009), Journal of Nursing Management	Examine factors evoking job satisfaction among nurses	M: Database search Y: 1995–2007 (varies slightly between databases) L: Not specified (English) S: Nurses working in hospitals O: Peer-reviewed, studies carried out in Western countries	21: 16 quantitative, 5 qualitative	Not specified beyond search approach Study details provided	United States (12), England (3), Canada (2), Norway (2), Finland (1), Australia (1)	Content analysis, inductive	• Interpersonal relationships • Human relationships with co-workers • Feeling of togetherness • Interaction and communication • Team work • Social climate and ethicality • Peer support • Patient care • Significance of patient care to nurses • Opportunity for high-quality patient care • Good human connections with patients • Ways of organizing work • Work-family-relationship • Supportive leadership • Work environment • Manageable and suitable workload • System of nursing practice • Salary and benefits
							• Variety of work • Autonomy • Professionalism and professional development
Vagharseyyedin (2016), Iranian Journal of Nursing and Midwifery Research	Integrate determinants of nurses' organizational commitment in hospital settings	M: Database and reference search Y: 2000–2013 L: English or Persian S: Hospital nurses O: Published in peer-reviewed journals, nurses results reported separately, experimental studies were excluded	33: 32 quantitative, 1 qualitative	AACN (American Association of Critical Care Nurses) revised evidence leveling system	Canada (7), Iran (6), Taiwan (6), United States (3), Australia (2), Belgium (1), China (1), Finland (1), Japan (1), Korea (1), Singapore (1), Mixed (3: Asian-American, EU, Malaysia England)	Thematic analysis	63 factors grouped into nine themes and four main categories: • Personal characteristics and traits of nurses • Biopsychosocial parameters • Personal and family life • Leadership and management style and behavior • Nature of relationships • Leadership style • Organizational context • Organization's norms and performance • Organizational policies and procedures • Characteristics of job and work environment • Growth and development, Content and organization of tasks • Mutual respect
Zhang et al. (2018), Applied Nursing Research	Conduct a meta-analysis on the relationship between structural empowerment, psychological empowerment and burnout for registered nurses.	M: database and manual search Y: from 1990 onward L: English or Chinese S: Registered nurses working in hospitals O: Quantitative cross-sectional studies reporting Pearson or Spearman correlation coefficients; results appraised by two reviewers	24: all quantitative	Quality In Prognosis Studies (QUIPS) tool by two reviewers Study details in table	China (11), Canada (6), United States (3), Egypt (1), Netherlands (1), Sweden (1), Turkey (1)	Meta-Analysis with data extraction by two reviewers	Structural empowerment

### Findings

Three key job demands and six job resources of nurses that are relevant across a broad range of health-related and motivational outcomes emerged from our analysis, as illustrated in Table 3.

**Table 3:** Identified key job demands and resources of nursing staff with represented aspects.

**DEMANDS**	
**Work Overload**	
• Workload	Hayes et al., 2010, 2012; Daouk-Öyry et al., 2014
• Work pressure, workload/time/staffing	McVicar, 2016
• Demand-control/effort-reward imbalance	Bernal et al., 2015
**Lack of Formal Rewards**	
• Pay/benefits/financial rewards/unequitable pay	Coomber and Barriball, 2007; Hayes et al., 2010, 2012; Daouk-Öyry et al., 2014; McVicar, 2016; Vagharseyyedin, 2016
• Growth and development opportunities	Hayes et al., 2012; Daouk-Öyry et al., 2014; McVicar, 2016
• Job security	Vagharseyyedin, 2016
• Effort-reward imbalance	Bernal et al., 2015
**Work-Life Interference**	
• Work-life or work-family conflict	Daouk-Öyry et al., 2014; McVicar, 2016
• Rostering/scheduling/shift work	Hayes et al., 2010; Keyko et al., 2016; McVicar, 2016
**RESOURCES**	
**Supervisor Support**	
• Supervisor support	Cowden et al., 2011; Hayes et al., 2012; McVicar, 2016
• Social support from supervisor/organization	Hayes et al., 2010, García-Sierra et al., 2016
• Organizational/management support	García-Sierra et al., 2016
**Fair and Authentic Management**	
• Authentic leadership	García-Sierra et al., 2016; Keyko et al., 2016
• Management: trust, fairness, respect	Germain and Cummings, 2010; Vagharseyyedin, 2016
• Supervisor incivility	Vagharseyyedin, 2016
• Organizational trust and fairness	Vagharseyyedin, 2016
**Transformational Leadership**	
• Transformational leadership	Cowden et al., 2011; Hayes et al., 2012; García-Sierra et al., 2016
• Leadership practices: vision, inspiration, mentoring	Germain and Cummings, 2010; Vagharseyyedin, 2016
**Interpersonal Relations**	
• Personal and professional interactions between employees or with other stakeholders	Utriainen and Kyngäs, 2009; Hayes et al., 2010; Daouk-Öyry et al., 2014; Keyko et al., 2016; McVicar, 2016; Vagharseyyedin, 2016
• Social climate/work climate, community	Utriainen and Kyngäs, 2009; García-Sierra et al., 2016
• Mutual respect/professional status	Hayes et al., 2010; McVicar, 2016; Vagharseyyedin, 2016
**Autonomy**	
• Autonomy	Germain and Cummings, 2010; Hayes et al., 2010; Keyko et al., 2016
• Control/skill discretion/decision latitude	Daouk-Öyry et al., 2014; Keyko et al., 2016; McVicar, 2016
• Demand-control	Bernal et al., 2015
**Professional Resources**	
• Professional practice environment/possibility for high-quality patient care	Utriainen and Kyngäs, 2009; Keyko et al., 2016
• Access to resources	Germain and Cummings, 2010
• Structure/organization of tasks and work	Daouk-Öyry et al., 2014; Vagharseyyedin, 2016

#### Key Job Demands

The first key job demand we identified was *work overload*. Work overload encompasses aspects of time pressure and staffing and revolves around the quantitative amount of work that needs to be done within a certain amount of time, rather than the quality of the tasks themselves. Another demand we identified was *lack of formal rewards*. The issue of pay was apparent in many of the included reviews and included satisfaction with and perceived fairness of pay. We categorized lack of formal rewards as a demand, as it appeared to be the lack thereof that was considered draining, rather than that an abundance of it could exert a greater motivational effect. The same can be said for lack of opportunities for career advancement, which is another important facet of this theme. The third demand that emerged from our analysis was that of *work-life interference*. This related predominantly to shift work and rostering and included aspects such as type of shift, number of hours worked and interference of work hours with non-work life.

#### Key Job Resources

The majority of the relevant workplace antecedents identified in our analysis were job resources. A glance at Table 2 confirms that most of the included individual reviews also report antecedents that could better be described as resources than demands.

The area that emerged most strongly from our analysis was that of leadership, so much so that it is reflected in three different job resources of nurses. We will address the distinction between management and leadership in the discussion below.

The first job resource is *supervisor support*, which was typically not further specified in the reviewed publications. In addition to that, we identified two distinct types of leadership/management style. The first one, which we call *fair and authentic management*, focusses on the perceived authenticity and fairness of managers, evoking trust in the employees. This includes, but is not limited to, one's immediate supervisor. The other one is *transformational leadership*, a style that is characterized by providing inspiration, guiding change, mentoring staff and following a participatory approach.

*Interpersonal relations* featured as a major job resource as well. This is relevant in interaction with nursing peers, but also with other groups of stakeholders, such as physicians, patients, their relatives, or other healthcare staff. Our findings suggest that the specific stakeholder group is of less relevance than the quality of the interactions. Mutual respect, support and appreciation contribute to a positive work climate and make social interactions resourceful. The importance of *autonomy* at work is widely acknowledged and featured prominently in our findings as well. It was identified in almost all included reviews. Autonomy comprises control over how to organize one's work and autonomy in making decisions, but also includes aspects of skill discretion. Lastly, *professional resources* was identified as the sixth key job resource for nurses. This encompasses those physical and organizational aspects that support the provision of high-quality nursing care.

## Discussion

In this integrative review of reviews, we examined the existing literature on workplace-level antecedents to a broad range of motivational and health-related outcomes in nursing staff with the aim of identifying the key job demands and resources across these outcomes. Our qualitative analysis identified three key job demands and six key job resources, namely work overload, lack of formal rewards, work-life interference, supervisor support, fair and authentic management, transformational leadership, interpersonal relations, autonomy and professional resources.

***Work overload*:** The demand of work overload we identified, was determined primarily by workload, time pressure and staffing. It is widely acknowledged that the workload for nurses has substantially increased in recent years and has reached unsustainable levels in many places. McVicar (2016) also identified a job demand called work pressure and emphasized the importance of workload, time and staffing. A study by Skinner and Pocock (2008) examining sources of work-life conflict, found that having too much work to do, was a stronger predictor of work-life conflict than time-related aspects such as number of work hours or control over work schedule, also highlighting the toll that excessive workloads take on employees.
Although not immediately obvious, work overload is also related to moral distress. Moral distress has been defined as an occurrence in which one knows the right action to take, but is constrained from taking it (Jameton, 1984). In a study examining moral distress among registered nurses in the United States, Zuzelo (2007) found that working with staffing levels that are considered unsafe, was the most morally distressing event out of 29 events that participants were asked to rate.
***Lack of formal rewards*:** Our findings illustrate the importance of pay and advancement opportunities for nursing staff, especially with regards to motivational outcomes. However, we gained the impression that pay was not a motivator, but rather that unfairness of pay, insufficient pay and also lack of advancement opportunities were perceived as adversarial. Coomber and Barriball (2007) reverberate this and refer to their included studies in which participants have expressed perceived unfairness of pay, especially given the high levels of education, experience and responsibility in nursing work. Both Coomber and Barriball (2007) and Hayes et al. (2012) point out that insufficient pay may more strongly affect turnover intention in male nurses than in female ones. This turnover intention, however, may also be strongly affected by the availability of suitable alternatives. While several of the included reviews note the importance of advancement opportunities, Hayes et al. (2012) remark that advancement opportunities seem to rank particularly highly among younger nursing staff.
***Work-life interference*:** Work-life interference may be particularly relevant to staff working shifts. From our findings, it appears that not only is shift work itself challenging, but also the rotating nature of shifts as well the limited plannability of spare time that nurses often experience. Work-life interference may also have particular relevance in nursing given the high percentage of women in that field who are disproportionately responsible for combining work and family responsibilities. Flinkman et al. (2010) review of 31 publications on nurses' intention to leave the profession found that family-work conflict was associated with higher intention to leave when it was inquired, however the authors point out that this aspect was rarely examined. Both McVicar (2016) and Keyko et al. (2016) acknowledge the importance of shift work and work-life interference. Keyko et al. group it under the physical and mental demands, while McVicar also identified a theme named work-life interference, which represents the facets of work-family conflict and shift working.
Three of the six key job resources for nurses that we identified relate to leadership, namely supervisor support, fair and authentic management and transformational leadership. The debate regarding the differences and overlap between management and leadership is ongoing. While management has more connotations of administration and dealing with the status quo, leadership seems more oriented toward change (Lunenburg, 2011). Maccoby (2000) described management as a necessary function, while leadership is about the relationship “between leader and led that can energize an organization” (Maccoby, 2000, p. 57). However, those functions may largely overlap (Nienaber, 2010). In face of these conceptualizations and the ongoing debate, we understand management and leadership to be different activities and functions that may be exercised by a person of authority in an organization, with management emphasizing administrative functions and leadership emphasizing change-oriented ones, and have named the job resources accordingly. While we analyzed the data inductively, we arrived at these three leadership-related resources which are already familiar from the literature and we find some of the established definitions to be good descriptors of the meaning of our themes also.
***Supervisor support*:** Findings related to this theme were typically either described in terms of support from supervisor, social support from peers and colleagues, or in terms of management/organizational support. However, what this support entails was typically not specified. The description of social support as emotional, instrumental, informational and appraisal support based on House (1981), seems to capture the meaning of our theme quite well. McVicar (2016) also reports a job resource of management and supervision support, while Keyko et al. (2016) did not identify such a resource.
***Fair and authentic management*:** The understanding of authentic leadership is predominantly shaped by Avolio et al. (2004, p. 802) view of authentic leaders as “persons who have achieved high levels of authenticity in that they know who they are, what they believe and value, and they act upon those values and beliefs while transparently interacting with others.” While some of the included reviews specifically referred to the concept of authentic leaderships, others, such as Germain and Cummings (2010) and Vagharseyyedin (2016) described leadership more generally in terms of fairness and trust.
***Transformational leadership*:** Transformational leadership was coined by Downton (1973) and Burns (1978) and today constitutes one of five components of the magnet model of the American Nurses Credentialing Center. Transformational leadership focuses on leading employees through change and emphasizes the importance of vision, influence and communication. Again, several of included reviews specifically examined this construct (Cowden et al., 2011; Hayes et al., 2012; García-Sierra et al., 2016), while others referred to the displayed leadership behaviors (Germain and Cummings, 2010; Vagharseyyedin, 2016).
Support for our results also comes from a systematic review of nine systematic reviews by Halter et al. (2017), who report managerial style—especially transformational leadership—and supervisory support as among the most relevant factors affecting nurse turnover. Findings from Gregersen et al. (2011, 2014) concerning the comparison of different leadership approaches, also lend support to the leadership-related resources we identified and provide insights into the possible relationships between them. Gregersen et al. (2011) literature review of employees in different industries linked leadership to employee well-being and found that social support was strongly associated with employee health, as were transformational leadership and employee-oriented leadership. Based on a subsequent empirical study with more than 1,000 nursing home employees in Germany (Gregersen et al., 2014) they propose that an important shared element between these different health-promoting leadership approaches may lie in trustful inter-individual relationships between the supervisor and the employee. This supports an individualized approach to interacting with each employee, rather than adhering strictly to a specific leadership style. The three leadership-related resources we identified could serve as pointers for positive leadership behaviors that need to be further tailored to fit each leader and employee. A need for differentiation is also illustrated by findings from Bringsén et al. (2012) who conducted a focus group study on workplace health resources among Swedish healthcare workers. They discovered that while a proportion of the participants associated health with flexibility at work, the others associated it with stability.
***Interpersonal relations:*** Our job resource of interpersonal relations refers to mutually respectful, supportive and appreciative relations between nurses and other stakeholders such as physicians, management, other healthcare staff, patients, and their relatives. Utriainen and Kyngäs (2009) emphasize the relevance of the general social climate for job satisfaction, while García-Sierra et al. (2016) focus on the impact of social support. Daouk-Öyry et al. (2014) also point out the negative impact of feeling undervalued or disrespected by colleagues or lack of collegiality. Aspects of professional status and mutual respect also play into this job resource. Interestingly, it appears to be the quality of the relationships rather than the specific stakeholder group that is more relevant. In a way this makes sense. Nurses act as a hub between many different stakeholder groups in patient care, among which physicians, for example, are only one group. Like us, neither McVicar (2016) nor Keyko et al. (2016), both of whom highlighted the importance of interpersonal relations, found enough evidence for a theme relating to interactions with a particular stakeholder group. As Aiken et al. (2013, p. 151) pointed out: “Nurses' relationships with physicians appear not to be as problematic as nurses' relationships with management.”
Interprofessional collaboration is a closely related concept and can be viewed from two different perspectives. The first, and in our impression more dominant one, relates to the coordination of care by different groups of healthcare professionals with the goal of reducing overlapping responsibilities and ensuring optimal continuum of care (WHO, 2010). The second perspective relates to the psychosocial interactions between different groups of professionals and includes aspects of mutual respect and recognition. In our findings, this second perspective also became apparent in our job resource of interpersonal relations.
***Autonomy*:** Most of the reviews included in our analysis recognized the importance of either autonomy (Germain and Cummings, 2010; Hayes et al., 2010), autonomy/control (Keyko et al., 2016), job control (Daouk-Öyry et al., 2014), decision latitude (McVicar, 2016) or demand/control (Bernal et al., 2015). Germain and Cummings (2010) also emphasize the importance of management in empowering autonomy among nurses. Our understanding of autonomy is closest aligned with Weston's description of clinical autonomy as “the authority and freedom of the nurse to make nursing care decisions concerning the content of clinical patient care in an interdependent practice” (Weston, 2008, p. 407). Autonomy has also been reported as a main job resource for the general working population (Bakker and Demerouti, 2007; Brauchli et al., 2015). A related concept that gained prominence in nursing is that of control over nursing practice. Control over this describes nurses' ability to shape departmental and organizational policies and practices related to nursing care (Weston, 2008), however this higher-order concept is predominantly located on the organizational level and looks beyond task design and individual decision-making.
***Professional resources*:** With professional resources, we refer to the immediate resources that support nurses in doing their work well. This includes tangible resources like work equipment but also intangible ones, such as access to necessary information and the organization of work tasks. This resource emerged as the vaguest result from our analysis. This may have been caused, at least in part, by the overlap with two other established concepts, professional practice environment and structural empowerment. Both concepts, however, are much broader in scope. Such broader themes of professional practice environment and professional practice and development have also been reported by Keyko et al. (2016), while McVicar's (2016) demand of physical resources was narrower.
Professional practice environment was one of the key findings that came out of the original magnet studies (McClure et al., 1983), when researchers conducted group interviews with nurses across the United States in hospitals that were known for being able to attract qualified staff despite a national nurse shortage. Aspects that were found to contribute to a professional practice environment were qualification and competency of nurses and managers, autonomy, and professional recognition among others. Structural empowerment of nurses focuses on organizational structures, policies and programs, opportunities for growth and visibility of nursing in the organization with the aim of empowering professional nursing practice and describes work environments that provide access to information, resources, support, and the opportunity to learn (Kanter, 1977). We view professional practice environment and structural empowerment as higher-order concepts that in fact include most of job resources we have identified. However, determining workplace attributes at the level of job resources and demands allows them to be more directly addressed on the team and leadership level. This is the reason why two of the review publications we included are not represented in Table 3. Cicolini et al. (2014) and Zhang et al. (2018) specifically examined the impact of structural empowerment on job satisfaction and burnout, respectively. However, the breadth of structural empowerment made them unsuited to fitting into our pattern of individual job demands and resources.
There are some notable similarities of our findings with established job demands and resources for the general working population, as well as differences to them. Our demand of work overload is comparable to Bakker et al. (2005) demand work overload as well as to Brauchli et al. (2015) demand time pressure, all of which emphasize quantitative aspects. Similarly, work-home interference (Bakker et al., 2005) resembles our work-life interference. Like our findings, all of the publications also acknowledge the importance of the job resources autonomy/control and support.
Separate physical, emotional, or physical demands (Bakker et al., 2005; Bakker and Demerouti, 2007) or work interruption, qualitative overload or uncertainty at work (Brauchli et al., 2015) did not emerge as key job demands from our analysis. While several of the included reviews specifically described emotional or physical demands in nurses, neither of these were reported consistently enough to form their own theme in our analysis. Had the included reviews involved more samples of nursing home staff, however, a different picture may have emerged, as physical demands have been found to be particularly high in that setting (Simon et al., 2005). Emotional demands featured strongly in McVicar's (2016) results, but also contained a sub-aspect of dealing with patients and relatives, which we grouped under interpersonal relations. It remains unclear whether emotional and physical demands constitute highly relevant job demands for nurses and what exactly constitutes emotional demands in nursing. Is it dealing with suffering and death, compassion fatigue (Joinson, 1992) or the emotional labor (Hochschild, 1983) of regulating ones' own emotions in the interactions with patients and their relatives? One might argue that these aspects are inherent to nursing and caring work and cannot be affected on the team or leadership level and that the focus should rather be on those workplace aspects that hinder nurses in coping with these demands (e.g., work overload) or support them in successfully doing so (e.g., social support).
Another aspect that could be closely related to emotional demands is the experience of aggression and violence, which was rarely included in the reviews we examined. Meanwhile, the health sector reports the highest percentage of workers who experience adverse social behaviors such as verbal abuse, physical violence, unwanted sexual attention, humiliating behavior, and harassment (Eurofound, 2017).
As illustrated in Table 2, studies from many different countries are reflected in our integrative review of reviews, although the majority stems from Northern America and Europe. A previous qualitative analysis of workplace stressors of nurses in five countries, including Hungary, Israel, Italy, the United Kingdom, and the United States found significant differences between the evaluation of different stressors but also showed similar patterns across the countries, which led the study authors to conclude that there are both emic (culture-specific) and etic (culture-general) sources to work stress in nurses (Glazer and Gyurak, 2008). Aiken et al. (2012) showed that although the degree to which nurses report burnout, job dissatisfaction or the intention to leave varied substantially between 13 surveyed countries (twelve European countries and the United States), these outcomes were associated with work environment aspects like staffing, managerial support, or participation in decision-making in all countries. This supports the potential for our findings to have an impact, even across different cultural settings.

### Limitations and Outlook

Several limitations need to be considered in interpreting our findings. First of all, although we intended to identify the relevant job resources and demands for both nurses and nursing assistive personnel working in both hospital and nursing home settings, the vast majority of studies included in the reviews we found were based on registered nurses in hospitals. This limitation needs to be considered when applying our findings to other nursing roles or work contexts, such as nursing homes, home care or assistive nursing personnel. Given the increasing importance of these roles and settings, the high demands placed on them and the lower levels of support often available to them (Kliner et al., 2017), future research should focus on examining workplace demands and resources in those contexts.

Next, only constructs that have been studied as antecedents and have found their way into reviews were reflected on our study. This implies that constructs which have thus far received limited attention in research, such as workplace violence, were underrepresented, while trending research topics, like transformational leadership, may have been overrepresented. Of course, this will have affected our findings.

The reviews in our analysis reflect studies conducted in many different countries and regions. Accordingly, our findings will have to be interpreted differently in different contexts. For example, career advancement opportunities for nursing staff may be very limited in some places, while in others, such as the United States, they are quite substantial. Many local aspects such as educational preparation of nursing staff, standards of care, labor regulations or social norms (see for example Hofstede, 1980) need to be considered when working with these job demands and resources.

Lastly, our findings should be further examined quantitatively to determine whether the proposed key job demands and resources do indeed explain more variance in health-related and motivational outcomes in nurses than do those identified for the general working population.

### Conclusion

The aim of our study was to identify the key job resources and demands of nursing staff by integrating findings from previously published review studies along the lines of the JD-R model. Our analysis revealed work overload, lack of formal rewards and work-life interference as the three key job demands of nursing staff. The key job resources were supervisor support, fair and authentic management, transformational leadership, interpersonal relations, autonomy and professional resources. In our analysis we considered a broad range of relevant health-related and motivational outcomes in nursing staff. With this integrative review of reviews, we were able to: (a) corroborate findings from previous reviews by McVicar (2016) and Keyko et al. (2016), (b) broaden the perspective beyond single outcomes on what makes workplaces motivating and health-promoting for nursing staff, which enhances the relevance and generalizability of our findings, and (c) illustrate the paramount importance of leadership practices in nursing. Understanding the most important job demands and resources can support the development of targeted interventions. This could occur on different levels, initiated either by nurse team leaders or managers or at the organizational level. As a first step, the use of established assessment tools can serve to determine to which extent the identified job demands and resources are present in a given work setting, based upon which steps to strengthening existing resources, building additional ones and reducing job demands can be implemented. For researchers, the understanding of the key job demands and resources specific to nursing staff supports the investigation of the relative importance of these different workplace-level antecedents to health-related and motivational outcomes.

## Author Contributions

The data analysis and manuscript were prepared by SB with support from GJ and GB. All authors critically reviewed and contributed to the manuscript and approved the final version.

### Conflict of Interest

The authors declare that the research was conducted in the absence of any commercial or financial relationships that could be construed as a potential conflict of interest.
